# Studying tree response to biotic stress using a multi-disciplinary approach: The pine pitch canker case study

**DOI:** 10.3389/fpls.2022.916138

**Published:** 2022-09-09

**Authors:** Joana Amaral, Luis Valledor, Artur Alves, Jorge Martín-García, Glória Pinto

**Affiliations:** ^1^Centre for Environmental and Marine Studies (CESAM), Department of Biology, University of Aveiro, Aveiro, Portugal; ^2^Department of Organisms and Systems Biology, University of Oviedo, Oviedo, Spain; ^3^University Institute of Biotechnology of Asturias, University of Oviedo, Oviedo, Spain; ^4^Department of Vegetal Production and Forest Resources, University of Valladolid, Palencia, Spain; ^5^Sustainable Forest Management Research Institute, University of Valladolid-INIA, Palencia, Spain

**Keywords:** physiology, omics, immune defence, plant pathogen, biotic stress, forest disease, control measures, *Pinus*

## Abstract

In an era of climate change and global trade, forests sustainability is endangered by several biotic threats. Pine pitch canker (PPC), caused by *Fusarium circinatum*, is one of the most important disease affecting conifers worldwide. To date, no effective control measures have been found for this disease. Earlier studies on PPC were mainly focused on the pathogen itself or on determining the levels of susceptibility of different hosts to *F. circinatum* infection. However, over the last years, plenty of information on the mechanisms that may explain the susceptibility or resistance to PPC has been published. This data are useful to better understand tree response to biotic stress and, most importantly, to aid the development of innovative and scientific-based disease control measures. This review gathers and discusses the main advances on PPC knowledge, especially focusing on multi-disciplinary studies investigating the response of pines with different levels of susceptibility to PPC upon infection. After an overview of the general knowledge of the disease, the importance of integrating information from physiological and Omics studies to unveil the mechanisms behind PPC susceptibility/resistance and to develop control strategies is explored. An extensive review of the main host responses to PPC was performed, including changes in water relations, signalling (ROS and hormones), primary metabolism, and defence (resin, phenolics, and PR proteins). A general picture of pine response to PPC is suggested according to the host susceptibility level and the next steps and gaps on PPC research are pointed out.

## Introduction: An overview of pine pitch canker

Forests cover nearly 31% of the total land area globally (4.06 billion ha), representing important economic, environmental, and social assets [Food and Agriculture Organization of the United Nations (FAO), 2020]. In particular, European forests are mainly composed of conifers (46%), with pine species being the greatest growing stock, key for CO_2_ sink and a vital source of resin and timber (Forest Europe-Ministerial Conference on the Protection of Forests in Europe, 2020). Forest sustainability is currently threatened by several biotic and abiotic factors that negatively affect its productivity and health. The vulnerability to these stresses is increased by climate change and the global trade of plants and derivates, especially regarding the importation of plant pathogens which may find further opportunities to colonise new environments (Sturrock et al., 2011; Santini et al., 2013; Wingfield et al., 2015).

**Figure 1:**
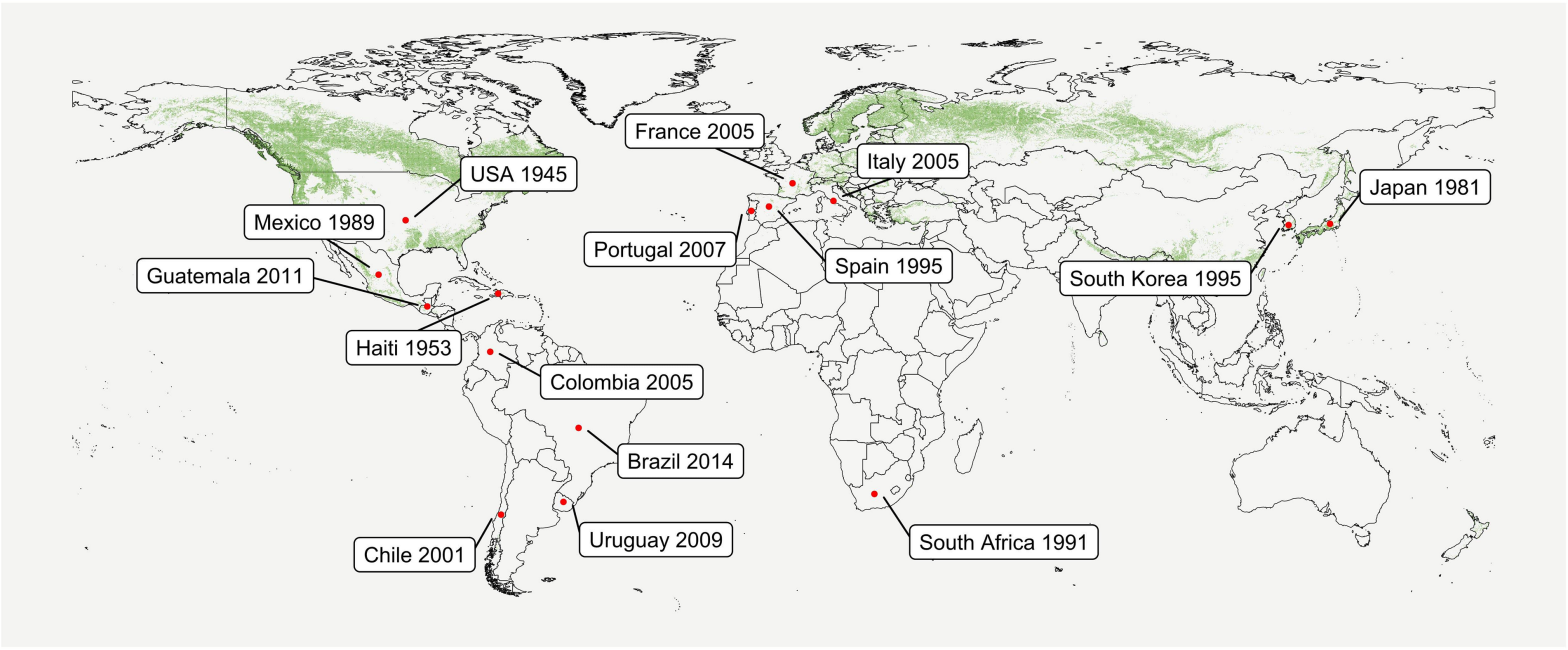
Conifer forest distribution and first reports of *Fusarium circinatum* worldwide. Green dots represent evergreen, deciduous, and mixed needle forests (min. area of 1 km^2^) according to the Corine Land Cover (CLC) 2012 v. 2020_20u1 provided by the European Union Copernicus Land Monitoring Service, European Environment Agency (EEA). Red points indicate areas in which PPC has been reported and the year of the first observation. This data have been recently reviewed by Drenkhan et al. (2020), where further detail regarding the sampling site (nursery or field), identification method, and host species can be found. The report of *F. circinatum* in Guatemala (Dvorak et al., 2011) was also included. Databases and figures were built using R v3.6.0.

Pine Pitch Canker (PPC), caused by the ascomycete fungus *Fusarium circinatum* Nirenberg & O’Donnell (sexual morph *Gibberella circinata* Nirenberg & O’Donnell), is one of the most important diseases affecting conifers worldwide (Wingfield et al., 2008). Although PPC was first reported in the United States (Hepting and Roth, 1946), phylogenetic analyses indicate that *F. circinatum* is originally an endemic member of the native Mexican forest (Wikler and Gordon, 2000). Introduction into Europe, Japan, and South Africa seems to have occurred through United States or Mexico (Wikler and Gordon, 2000; Berbegal et al., 2013). PPC has now been reported globally (Figure 1; reviewed by Drenkhan et al., 2020). As the natural spread of *F. circinatum via* rain, wind, and insects is limited by short spore dispersal and vector-insect flight distances, longer distance dissemination is mainly human-assisted (Zamora-Ballesteros et al., 2019). This occurs mainly through the movement of infected seeds worldwide, and of asymptomatic seedlings, and contaminated substrates, or containers regionally.

After a new introduction occurs, moisture and warm temperatures are crucial for the establishment and spread of PPC (Wingfield et al., 2008; Ganley et al., 2009; Baker et al., 2010; Quesada et al., 2019; Elvira-Recuenco et al., 2021). A large area of European pine forests has been reported to be suitable or optimal for PPC development, with *F. circinatum* distribution potentially changing towards northern Europe due to the predicted reduction of cold and drought (Ganley et al., 2009; Baker et al., 2010; Watt et al., 2011; Möykkynen et al., 2015). This is of particular concern giving that *F. circinatum* showed to be able to infect conifers present in these currently disease-free areas such as *Pinus sylvestris* L. and *Picea abies* (L.) H. Karst (Martín-García et al., 2017, 2018), and that these areas are populated with a high density of conifers (Figure 1). In nurseries, optimal conditions may be easily achieved for disease development in imported infected seeds or plants independently of its location (Drenkhan et al., 2020).

Pine pitch canker affects more than 60 species of *Pinus* and *Pseudotsuga menziesii* (Mirb.) Franco (Bezos et al., 2017) at all stages of tree development infecting seeds, seedlings, and mature trees [European and Mediterranean Plant Protection Organization (EPPO), 2021]. Disease symptoms include excessive pitch flow, resinous canker formation, and branch/canopy dieback in adult trees and tip dieback and seedling damping-off in nurseries (Wingfield et al., 2008; Martín-Rodrigues et al., 2013; Santana et al., 2016). These results in significant economic losses in nurseries and pine plantations/stands (Aegerter et al., 2003; Wingfield et al., 2008; Carrasco et al., 2016). Although natural infections occur through wounds (Iturritxa et al., 2011), the pathogen is also able to colonise seedlings or roots of mature trees without damaging the adjacent tissues allowing plants to remain asymptomatic (Elvira-Recuenco et al., 2015; Swett et al., 2016, 2018; Martín-García et al., 2018; Hernandez-Escribano et al., 2018a). This hemibiotrophic nature represents an additional challenge to manage PPC (Vettraino et al., 2018); as well as its ability to naturally infect grasses surrounding pine species, where it may represent a source of inoculum (Swett and Gordon, 2012; Swett et al., 2014; Hernandez-Escribano et al., 2018b; Herron et al., 2020).

Several studies focused on *F. circinatum* infection mechanism and virulence. After conidia germination, the pathogen colonises the host stem radially advancing towards the pith, likely releasing cell wall degrading enzymes (e.g. endopolygalacturonase) to liberate nutrients from the host tissues (Chimwamurombe et al., 2001; Martín-Rodrigues et al., 2013). Vertical colonization of the host occurs later through the spread of conidiophores and conidia when the pathogen reaches the phloem or the traumatic resin ducts in the xylem (Martín-Rodrigues et al., 2013). Physical obstruction by *F. circinatum* growth, generalised cell death in the xylem and increased resin production may restrict water supply and result in plant death (Gordon, 2011; Martín-Rodrigues et al., 2013). Different phenotypical and molecular characteristics have been associated to *F. circinatum* pathogenicity or virulence (Mullett et al., 2017; Muñoz-Adalia et al., 2018a,b; Phasha et al., 2021a). Also, several compounds have been proposed to allow host colonization, including beauvericin, ergosterol, fusaric acid, and laccases (Fotso et al., 2002; Muñoz-Adalia et al., 2016; Visser et al., 2019; Phasha et al., 2021b).

In general, earlier research on PPC was focused on the characterization of *F. circinatum* populations and on testing the susceptibility of different hosts to PPC, with information on the mechanisms behind these differential responses remaining scarce. Over the last few years, a new perspective has gained relevance aiming to unveil these mechanisms based on an integrative approach (from physiology to Omics). This review highlights the main advances on PPC knowledge, with a special focus on multi-disciplinary studies exploring host response to *F. circinatum* and on how these contribute for tree biotic stress response knowledge and for the development of effective disease control measures.

## PPC resistance: Exploiting genetic variance using an integrated approach, from physiology to omics

Exploring genetic resistance based on intra- and inter-specific variation has been proposed as a promising environmentally-friendly strategy to avoid the natural spread of PPC (Martín-García et al., 2019; Zamora-Ballesteros et al., 2019). This would allow for the selection/development of resistant trees to reforest high-risk sites. Host susceptibility to *F. circinatum* is known to vary at the species, population, family, and clone levels (reviewed by Drenkhan et al., 2020). While some species, such as *Pinus radiata* D. Don and *Pinus patula* Schltdl. & Cham., are highly susceptible to *F. circinatum,* others are moderately susceptible (*P. pinaster* Aiton) or resistant (*P. pinea* L.). *Pinus radiata* has been extensively studied as it is the most planted pine worldwide due to its fast growth and wood quality (Mead, 2013). The among-population variation found in some species has been attributed to geographical and environmental gradients (e.g., resistance in low elevation *Pinus tecunumanii* Eguiluz & J. P. Perry). Moreover, variation in PPC resistance showed to be quantitative and dependent on polygenic mechanism (i.e. results from the integration of small effects of several genes; information mainly based on SNPs associations; Gordon et al., 1998; Kayihan et al., 2005; Quesada et al., 2010), and presents variable heritability estimates (reviewed by Martín-García et al., 2019).

Although resistance to diseases in forest trees has been mostly achieved based on phenotypic selection, advances on genomic techniques and resources contributed for the identification of candidate genes related to biotic stress tolerance (Kovalchuk et al., 2013; Muranty et al., 2014; Sniezko and Koch, 2017). For example, the integration of multiple Omics level data from trees with varying levels of susceptibility to the disease may be used to identify candidate genes or metabolic pathways for manipulation (Naidoo et al., 2019). Innovative bioinformatic and statistical tools allow to build networks that model the dynamics and complexity of a given biological system linking molecular interactions and complex traits (zu Castell and Ernst, 2012; Valledor et al., 2018; Naidoo et al., 2019), such as PPC. This systems biology view may provide foundation for defining specific gene sets relevant for PPC resistance. Despite possible legislative barriers, these may be targeted by novel gene editing tools if the desired combination of genes cannot be achieved through classical breeding (Chang et al., 2018).

**Figure 2:**
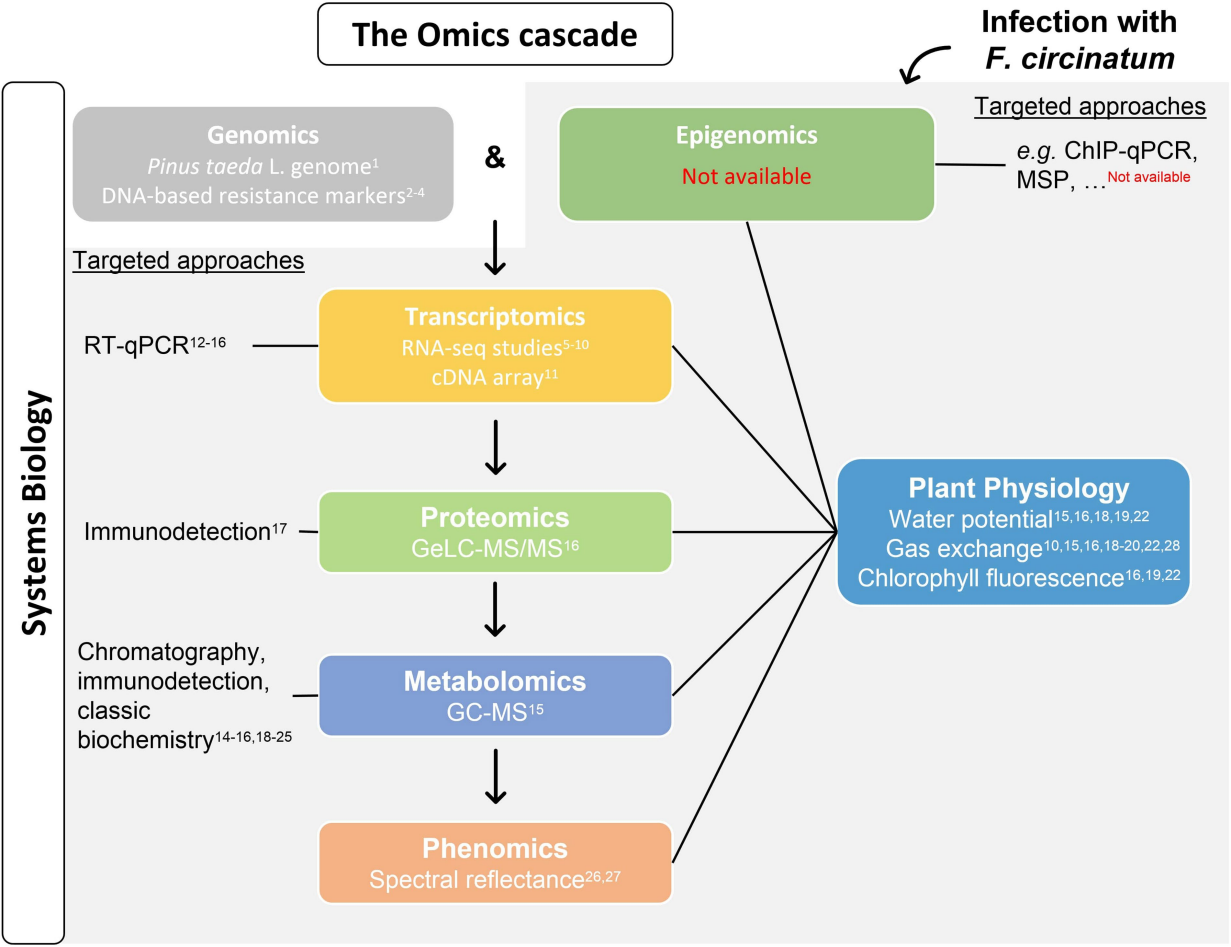
Advances and knowledge gaps on Pine pitch canker (PPC) research regarding the different levels of the Omics cascade. The availability of *P. taeda* genome is a useful tool for exploring *Pinus*-*Fusarium circinatum* interaction and allowed to identify DNA markers that determine the resistance of a certain genotype to PPC. In addition, *F. circinatum* infection induces changes at the DNA, RNA, and protein and metabolite levels, ultimately resulting in different phenotypes (grey background). While epigenetic studies were not performed on PPC, several transcriptomics studies included hosts with different susceptibilities and overtime analysis near the inoculation point. The single proteomic and metabolomic analyses available were performed using needles of pines with contrasting responses to *F. circinatum* infection. Phenomics studies are also available. Changes on the abundance of putative transcripts, proteins, and metabolites of interest have been explored and may complement/validate Omics data together with plant physiology measurements. The integration of this data provides a systems biology view of pine response to PPC. References: ^17^Davis et al. (2002), ^11^Morse et al. (2004), ^24^Kim et al. (2010), ^2^Quesada et al. (2010), ^12^Fitza et al. (2013), ^1^Neale et al. (2014), ^21^Vivas et al. (2014a), ^13^Donoso et al. (2015), ^5–7^Visser et al. (2015, 2018, 2019), ^28^López-López et al. (2016), ^26^Reynolds et al. (2016), ^8^Carrasco et al. (2017), ^22^Cerqueira et al. (2017), ^3^Lu et al. (2017), ^23^Silva-Castro et al. (2018a), ^27^Lee and Lee (2019), ^15,16,18,19^Amaral et al. (2019a,b, 2021a,b), ^4^De La Torre et al. (2019), ^25^Lombardero et al. (2019), ^9^Hernandez-Escribano et al. (2020), ^14^Otero et al. (2020), ^20^Leitão et al. (2021), and ^10^Zamora-Ballesteros et al. (2021). GC–MS, gas chromatography coupled to mass spectrometry; GeLC-MS/MS, gel-enhanced liquid chromatography–tandem mass spectrometry; RT-qPCR, reverse transcription quantitative PCR; ChIP-qPCR, chromatin immunoprecipitation-qPCR; and MSP, methylation-specific PCR.

The use of high-throughput Omics technologies in forest trees has lagged behind that of model or crop species, especially due to trees long-life cycles, large genome sizes, and the lack of genomic tools (Neale and Kremer, 2011). The availability of *Pinus taeda* L. whole-sequenced genome allowed for the identification of several single nucleotide polymorphisms (SNPs) associated with PPC resistance (Quesada et al., 2010; Lu et al., 2017; De La Torre et al., 2019; Figure 2). Moreover, the increasing performance and cost reduction of next-generation sequencing technologies make whole-transcriptome sequencing (RNA-Seq) attractive for the evaluation of gene expression in organism for which genome sequencing is not available, in particular for species with megagenomes such as conifers (e.g. Santos et al., 2012). Also, dual RNA-Seq strategies may address changes in both the host and the pathogen transcriptomes at once allowing to better decipher its interaction (Naidoo et al., 2018). RNA-Seq and dual RNA-Seq studies have been performed in pine species with different levels of susceptibility to PPC to unveil host responses to *F. circinatum* infection and explore how the pathogen is able to overcome them; as well as target transcriptomic studies (check Figure 2 for references).

However, changes at the transcriptional level are not always reflected at the downstream levels of the Omics cascade. Proteomics allow to study the proteins regulating disease development, contributing for the understanding of complex host defence mechanisms (Kaur et al., 2017; Rustagi et al., 2018). Although several of the proteomic studies in forest trees are based on two-dimensional electrophoresis (2-DE) coupled to MS analysis, resulting in low proteome coverage, forest proteomics have evolved, especially regarding LC–MS-based systems for protein identification and quantification using shotgun/bottom-up proteomics (Rodrigues et al., 2021). Early proteomics studies regarding PPC focused only on the role of a particular protein family (chitinases) in response to *F. circinatum* infection (Davis et al., 2002); while recent studies explored the needle proteome of pine species with contrasting PPC response phenotypes followed by integration with physiological data (Amaral et al., 2021b).

In addition, metabolites, at the end of the Omics cascade, represent a phenotypic signature of genetic variance, and of epigenetic, transcriptomic, and proteomic changes (Fiehn, 2002). As for proteomics, advances in MS approaches currently allow a large-scale profiling of plant metabolites (Rodrigues et al., 2021), allowing to identify biomarkers of forest diseases (e.g. Hantao et al., 2014; Wu et al., 2015) or screen disease resistant trees for breeding programs (e.g. Sambles et al., 2017; Wang et al., 2017). However, to distinguish whether the metabolites analysed are of plant or of pathogen origin may represent a great challenge. Unlike transcripts and proteins, metabolites identification does not depend on known genetic sequences. While some techniques have been proposed to address this issue, these are still associated to several drawbacks (see Tenenboim and Brotman, 2016). Regarding PPC, the quantification of specific metabolites, such as sugars, proline, hormones, terpenoids, phenolics, or lignin has been carried out using mainly traditional biochemical methods (check Figure 2 for references). GC–MS analysis of the needle primary metabolism of pines with different levels of susceptibility to *F. circinatum* infection were only recently performed together with physiological and targeted gene expression measurements (Amaral et al., 2019a).

Despite phenomics approaches are usually scarce in forest trees, some advances have been reported for PPC (Reynolds et al., 2016; Lee and Lee, 2019). Phenomics combine well-established plant physiology methodologies (e.g., gas exchange, chlorophyll fluorescence, water relations, nutrient uptake, and growth rate measurements) with recent technological advances to develop high-throughput phenotyping capture technologies, such as unmanned aerial vehicle remote sensing, to obtain a large-scale physiological overview of plants under different environmental scenarios (Flexas and Gago, 2018; Naidoo et al., 2019). The integration of different Omics levels with plant physiology theoretical knowledge and techniques is expected to maximise the understanding of plants functioning (Flexas and Gago, 2018); especially given that plant physiology has also proven useful for the understanding of plant-pathogen interaction (Berger et al., 2007). Studying the mechanism behind *Pinus*-*F. circinatum* interaction in hosts with varying levels of susceptibility to PPC based on a multidisciplinary approach unveiled defence response associated to each phenotype and is predicted to support innovative disease control measures.

## Host response to pathogen infection: State of the art on PPC

Plants possess a complex innate immune system that enables them to respond to pathogen attack, including non-specific constitutive defences (e.g., lignosuberised trees outer bark, needles cuticular surface, and phytoanticipins, such as phenolics, terpenoids, and alkaloids) and induced defences (Kovalchuk et al., 2013). The latter may be activated by the recognition of Pathogen-Associated Molecular Patterns (PAMPs) by extracellular domains of membrane receptor proteins (pattern-recognition receptors—PRRs) and result in PAMP-triggered immunity (PTI; Chisholm et al., 2006; Jones and Dangl, 2006; Nishad et al., 2020). The activation of PTI in response to *F. circinatum* infection in *P. pinaster* has been proposed due to the early upregulation of genes encoding a lysine motif receptor-like kinase (LysM-RLK), which has been associated to the recognition of fungal chitin (Hernandez-Escribano et al., 2020). Several genes coding for PRR containing domains have also been upregulated in the resistant *P. pinea* but not in the susceptible *P. radiata*, which may explain the weak downstream signalling in the latter (Zamora-Ballesteros et al., 2021). Regarding effector-triggered immunity (ETI), a second line of defence activated by the recognition of fungal effector proteins by plant resistance (R) proteins (Hammond-Kosack and Parker, 2003), there is still no evidence of its role in PPC.

After pathogen recognition, plants activate several defence responses to avoid its spread. These include morphological modifications (e.g., cytoskeletal reorganization and cell wall fortification); the production of reactive oxygen species (ROS) and secondary metabolites (such as phytohormones, phytoalexins, and plant volatiles), which may interact directly with the pathogen or function as signalling molecules distant from the infection site (Bolton, 2009; Eyles et al., 2010; Rojas et al., 2014); and the induction of pathogenesis-related (PR) proteins (van Loon et al., 2006) and a programmed cell death mechanism known as hypersensitive response (HR; Coll et al., 2011). The production of PR proteins in uninfected tissues is associated to the development of systemic acquired resistance (SAR), a systemic induced resistance (SIR) phenomena mediated by salicylic acid (SA) that protects plants against future infections (Fu and Dong, 2013). SIR is known to occur in trees in response to necrotrophic pathogens, including *F. circinatum*, although there is no sufficient information on the signalling pathways involved to identify the specific mechanism developed (Bonello et al., 2001, 2006; Eyles et al., 2010; Gordon et al., 2011). The use of chitosan as an elicitor of pine defence against *F. circinatum* has showed promising results based on the systemic induction of host resistance (e.g., activation of the phenylpropanoids pathway) and/or its fungistatic effect (Reglinski et al., 2004; Fitza et al., 2013; Silva-Castro et al., 2018a,b). The potential of using biotic (pathogens or beneficial microbes) and abiotic (e.g. wounding and chemicals) elicitors to trigger the establishment of induced responses in pines and enhance resistance to *F. circinatum* has been reviewed by Martín-García et al. (2019). Promising promoters of PPC resistance may be part of an integrated management approach to control PPC in an environmentally friendly manner.

A general reprogramming of host’s metabolism occurs to fuel plant defence responses against pathogens instead of growth, reproduction, and yield; but also to meet the nutritional requirements of the pathogen (accumulation of C and N sources; Berger et al., 2007; Bolton, 2009; Fagard et al., 2014; Rojas et al., 2014). For example, transcriptomic studies evidenced this metabolic trade-off between growth and defence in the *P. pinaster*-*F. circinatum* interaction: genes related to growth, morphogenesis, and photosynthesis were downregulated; while genes involved in phytohormone signalling, ROS regulation, oxidative stress, regulation of cell death, signal transduction, synthesis of flavonoids, anthocyanins, and other defence-related secondary metabolites were increased (Hernandez-Escribano et al., 2020). However, the late production of secondary metabolites was insufficient to fight pathogen infection.

The main aspects of pine response to *F. circinatum* are integrated in the following sections, including changes in plant water relations, signalling pathways (ROS and hormones), primary metabolism, and induction of common conifer defences (resin, phenolics, and PR proteins). These advances were achieved fusing techniques from different disciplines (from physiology to Omics) to explore changes in hosts with different susceptibility to PPC.

### Changes in plant water relations

The impact of canker development after *F. circinatum* infection in hydraulic failure and consequent tree death is well-documented. The dieback observed from the tips of pine branches to the *F. circinatum* infection site was shown to occur as a result of girdling cankers and pathogen growth obstructing water flow (Gordon et al., 2001; Martín-Rodrigues et al., 2013). Earlier studies demonstrated that the wilting preceding this dieback correlated in fact with the hampering of the water flow through stem segments showing similar canker sizes in *P. taeda*, although the possible effect of the phytotoxins produced by the pathogen has not been excluded (Solel and Bruck, 1990). These could include, e.g., beauvericin or fusaric acid which have shown to contribute to *F. circinatum* pathogenicity (Muñoz-Adalia et al., 2016; Phasha et al., 2021b).

At the molecular level, a SNP was located on a PPC resistance-related gene in *P. taeda* (*dehydration-responsive protein RD22-like*), suggesting that water deficit resistance was also induced as part of the defence mechanism against *F. circinatum* infection (Lu et al., 2017). The upregulation of *pi307a* (usually associated to drought responses) in *Pinus elliottii* Engelm. var. *elliottii* was suggested to reflect the shoot desiccation observed being an example of a “molecular symptom” (Morse et al., 2004). The authors propose that the obstruction/destruction of the vascular system by the pathogen results in the loss of solute transport, inducing a drought response in the upper parts of the shoot including the regulation of drought-responsive genes. A gene involved in stomatal closure was also found to be upregulated in *P. patula* seedlings only 1 day post-inoculation with *F. circinatum* (Visser et al., 2015).

Changes in plant water relations associated to PPC showed to modulate foliar gas exchange through stomata regulation. Physiological measurements revealed a decreased water potential coupled to a reduction of needle transpiration rate and stomatal conductance in symptomatic *P. radiata* and *P. pinaster* (Cerqueira et al., 2017; Amaral et al., 2019a,b, 2021a). However, the water stress-like scenario observed showed to be more intense for *P. pinaster* (lower water potential, decreased relative water content, overexpression of the drought-responsive *SnRK2.6* and *PR5*, and accumulation of the osmolyte proline; Amaral et al., 2019a,b). The greater resistance to PPC observed in *P. pinaster* has thus been attributed to its greater tolerance to water-limiting conditions in comparison with *P. radiata*, which would explain the earlier development of PPC symptoms at lower thresholds of water deficit in the highly susceptible species. This observation paves the way for exploring shared mechanisms underlying pine resistance to both drought and PPC and offers an opportunity to develop trees resistant to multiple stresses, which is of utmost importance in the current climate change context.

In contrast with the susceptible *P. radiata*, which opens its stomata immediately after infection with *F. circinatum* closing them after 1 day, the resistant *P. pinea* maintains this response overtime (Amaral et al., 2021a). Although the involvement of the glutathione-ascorbate cycle in this process has been proposed (Amaral et al., 2019a), this hypothesis has been excluded after enzymatic activity analysis (Amaral et al., 2021a). The role of stomata opening/closure in plant immune defence is well-described regarding foliar bacterial pathogens which use them as entering points for infection, but little is known about stomatal defence in pathogens that enter the host through other vias. This represents an exciting field of research as it could represent, e.g., a resistance mechanisms that relies on the maintenance of host photosynthetic capacity to avoid carbon starvation after pathogen infection and/or a strategy to optimise water uptake by decreasing needle water potential to compensate transpiration as ‘water spender’ species do under drought.

### Signalling mechanisms in the response to pathogen infection

#### ROS signalling, oxidative damage, and antioxidant mechanisms

In addition to its direct antimicrobial activity, many of the defences induced by pathogen invasion are associated to the rapid production of ROS (the ‘oxidative burst’; O’Brien et al., 2012). These include the induction of the expression of defence genes, HR, cell wall protein cross-linking, phytoalexin production, callose deposition, SAR, and the inactivation of key photosynthetic genes. However, at high levels, ROS may become toxic and interact with several organic molecules (e.g. proteins, nucleic acids, lipids, and carbohydrates) leading to oxidative-induced damages (Sharma et al., 2012; Soares et al., 2019). Therefore, plants have developed a complex antioxidant system composed of enzymatic and (low-molecular weight) non-enzymatic elements that help maintain cell redox homeostasis (Soares et al., 2019).

Several studies reported the involvement of these mechanisms in PPC. In *P. radiata*, (i) proline accumulation may explain unchanged malondialdehyde and electrolyte leakage values—indicators of cell membrane integrity—as proline protects proteins and membranes from denaturation and degradation and is linked to ROS scavenging (Cerqueira et al., 2017) and (ii) a long non-coding RNA (lncRNAPiRa.19024.1) seems to be involved in cell detoxification processes after an oxidative burst is triggered by *F. circinatum* infection (Zamora-Ballesteros et al., 2022). For relatively resistant species it is known that: (i) *P. pinaster* seedlings originated from a favourable maternal environment presented an effective antioxidant capacity which allowed to limit oxidative damage (necrosis) and *F. circinatum* growth (Vivas et al., 2014b); (ii) non-enzymatic components, such as L-ascorbate, glycerol, and vitamin B6 were associated to PPC resistance (Amaral et al., 2019a, 2021b; Zamora-Ballesteros et al., 2021); and (iii) the induction of phenolic compounds seems key in pine antioxidant response to *F. circinatum* infection (further discussed). The exogenous application of some of these natural compounds or others that induce its biosynthesis could be further explored for the development of environmentally friendly measures to control PPC.

Although the activity of important antioxidant enzymes from the ascorbate-glutathione and water–water cycles was not altered after *F. circinatum* infection in needles of the resistant *P. pinea*, the water–water cycle is compromised in symptomatic *P. radiata* (Amaral et al., 2021a). The failure of this photoprotective mechanism to dissipate the excess excitation energy generated after photosynthesis impairment may explain the increased susceptibility to photoinhibition observed, which suggests oxidative damages in PSII. Proteomic studies further discussed the importance of chloroplastic redox regulation in pine response to PPC (Amaral et al., 2021b). While in *P. radiata* it is hypothesised that the pathogen may target chloroplastic proteins for the redox regulation of key metabolic pathways, in the resistant *P. pinea* some chloroplastic proteins have been identified as part of its antioxidant response upon infection (chloroplastic peroxiredoxin Q and nucleoside diphosphate kinase 2). However, further studies are needed to better understand these mechanisms.

#### Regulation of hormone signalling pathways

Phytohormones are important signalling components of plant defence, establishing a complex crosstalk in response to infection depending on the plant-pathogen interaction (Marzec, 2016; Huang et al., 2020). However, fungal pathogens also have the ability to produce hormones contributing to plant disease (Chanclud and Morel, 2016). Interestingly, gibberellins (GAs; well-known growth-promoting phytohormones) were named after *Gibberella fujikuroi* (Sawada) S. Ito (= *Fusarium fujikuroi* Nirenberg) in which these were first identified (Stępień et al., 2020). *Fusarium circinatum* is part of the *F. fujikuroi* species complex and is thus also able to produce GAs (Stępień et al., 2020) although it possesses only one gene from the gene cluster related to its biosynthesis found in other *Fusarium* species (Bömke and Tudzynski, 2009). Besides fungal GAs are suggested to participate in pathogenicity (Chanclud and Morel, 2016), its role in PPC has not yet been determined.

On the other hand, the importance of hormone-mediate defence responses against *F. circinatum* infection has been highlighted by recent transcriptomic studies in different pine species (Carrasco et al., 2017; Visser et al., 2019; Hernandez-Escribano et al., 2020; Zamora-Ballesteros et al., 2021). While abscisic acid (ABA) signalling-related genes were part of the second most relevant category of genes differentially expressed in *P. radiata* upon inoculation (Carrasco et al., 2017) and ABA catabolism seems to be activated (Zamora-Ballesteros et al., 2021); a complex integration and coordination of SA, jasmonic acid (JA), and ethylene (ET), and auxins signalling is suggested to be involved in the resistance of *P. tecunumanii* and *P. pinea* to PPC (Visser et al., 2019; Zamora-Ballesteros et al., 2021), as well as the moderate resistance shown by *P. pinaster* at early stages of infection (Hernandez-Escribano et al., 2020). In particular, lncRNAPiRa.85000.6 has been suggested as a key regulator of ET levels after infection: while *P. pinaster* presents a fine-tuned ET response against *F. circinatum*, this does not occur in *P. radiata* likely due to the influence of this lncRNA located upstream of its transcription (Zamora-Ballesteros et al., 2022).

The initial response of *P. pinaster* has been suggested to be then manipulated by the ability of the pathogen to: stop SA biosynthesis through the chorismate pathway by the synthesis of isochorismatase family hydrolase (ICSH); block JA signalling by the suppression of a key regulatory element of this pathway, the receptor coronatine insensitive 1 (COI1), and perturb ET homeostasis by the production of fungal ET which can act as a virulence factor and interfere with ET signalling to suppress effective defence pathways (Hernandez-Escribano et al., 2020). However, the authors acknowledged that further physiological measurements (including hormone quantification) and functional studies with *F. circinatum* mutants are needed. The blocking of JA signalling by *F. circinatum* has been also proposed by Zamora-Ballesteros et al. (2021). Moreover, besides increased levels of JA were found in *P. radiata* and *P. pinaster* needles, these species were still susceptible to *F. circinatum* infection (Amaral et al., 2019a,b). This further supports that the pathogen can overcome JA-dependent defence responses.

Different studies report an increase of the endogenous levels of ABA in needles of *P. radiata* and *P. pinaster* upon inoculation with *F. circinatum* associated to stomata closure and photosynthesis impairment (Cerqueira et al., 2017; Amaral et al., 2019a,b, 2021a). Again, the authors discuss that this may result either from the host defence response or from its manipulation by the pathogen to suppress plant basal resistance. In accordance, lower levels of ABA were verified when a delay on disease symptom development was induced by the foliar application of phosphite (Cerqueira et al., 2017). The catabolism of ABA is also activated early in the infection process leading to the accumulation of the inactive dihydrophaseic acid in *P. radiata* (to control de pool of bio-active ABA) and of the weakly ABA-like active phaseic acid in the resistant *P. pinea*, which may be a key defence mechanism (Amaral et al., 2021a). Proteomic studies further suggest an ABA-mediated epigenetic reprogramming of gene expression, although this still lacks validation (Amaral et al., 2021b).

Despite the effort to untangle the intricate regulation of hormone balance during *Pinus*-*F. circinatum* interaction and understand how the pathogen may interfere with it has contributed to elucidate these signalling networks, there is still a long way towards the development of hormone-based control strategies for PPC. The constitutive activation of particular hormone signalling pathways (in mutants or transgenic lines) may enhance resistance to a certain pathogen but also impact plant fitness and increase susceptibility to other stresses (Denancé et al., 2013). A promising alternative to this would be “priming,” i.e., treat plants with a defensive compound, such as an hormone, in order to modulate its “immunological memory” so that a faster and stronger resistance response occurs upon attack (Conrath, 2011). The exogenous application of both SA and methyl jasmonate (MeJA) failed to enhance resistance to PPC (Vivas et al., 2012; Fitza et al., 2013). Given the relevance of ABA signalling in PPC outcome, the next step would be to explore the potential of ABA and its catabolites as inducers of resistance; as well as the chemical manipulation of key control points in ABA signalling, as this has been pointed out as an innovative approach to manage stress in agriculture (Hewage et al., 2020).

### Rearrangement of host primary metabolism: Photosynthesis, carbohydrates, and nitrogen

RNA-Seq analysis of two contrasting *P. radiata* genotypes in their response against *F. circinatum* revealed that the most interesting differentially expressed genes included genes related to primary metabolism (Carrasco et al., 2017). In general, it is known that the high demands of energy for defence response upon biotic challenge are associated with a reprogramming of the host primary metabolism, including changes in photosynthesis, carbohydrate and N metabolism, plant respiration, and aerobic fermentation (Berger et al., 2007; Bolton, 2009; Rojas et al., 2014).

Most photosynthesis-related studies in PPC report photosynthesis limitations at late stages of disease in susceptible hosts, likely due to oxidative damages in the photosynthetic apparatus. The expression of photosynthetic genes was decreased in the stem of the resistant *P. pinea* but not in *P. radiata*, although no changes on needle photosynthetic rate were verified (Zamora-Ballesteros et al., 2021). However, *RuBisCO* was downregulated in needles of symptomatic *P. radiata* and *P. pinaster* and independent gas exchange measurements revealed photosynthesis impairment in these species due to stomatal and non-stomatal processes, either maintaining or increasing its total chlorophyll content (López-López et al., 2016; Cerqueira et al., 2017; Amaral et al., 2019a,b, 2021a). Decreased photochemical efficiency (F_v_/F_m_ – maximum quantum yield of PSII and Φ_PSII_ – effective quantum yield of PSII) was also reported in *P. radiata* (Cerqueira et al., 2017), as well as increased susceptibility to photoinhibition (Amaral et al., 2021a).

Photosynthesis decrease may induce a metabolic shift towards respiration (from source-to-sink) through the transformation of the stored sucrose into fructose and hexoses (Berger et al., 2007; Bolton, 2009; Rojas et al., 2014). Sugars and its phosphate derivates are also key signalling molecules in several defence-related pathways (Morkunas and Ratajczak, 2014). Besides Cerqueira et al. (2017) did not found significant changes in *P. radiata* total soluble sugars content after *F. circinatum* infection; Vivas et al. (2014a) highlighted the role of carbohydrates in *P. pinaster* response to PPC, with seedlings originated from mother trees growing on a favourable environment showing greater tolerance to the disease and an altered carbohydrate type proportion (increased glucose vs. decreased uronic acids content). The authors suggested the involvement of epigenetic mechanisms in transmitting carbohydrate changes to offspring and highlighted the influence that the inherited endophyte community may have, but no further studies are available. Moreover, two genes linked to sugar metabolism were shown to be upregulated after inoculation of *P. patula* seedlings at very early stages of infection (Visser et al., 2015), and the downregulation of RuBisCO in symptomatic *P. radiata* and *P. pinaster* has been associated to a source-to-sink metabolic shift which was reflected in the induction of several alternative energy-producing pathways (e.g. respiration, aerobic fermentation, and gluconeogenesis/glyoxylate cycle) and in the accumulation of amino acids (Amaral et al., 2019a, 2021b).

Strong changes on host N metabolism are expected after pathogen infection as a result of the activation of defence response and of fungal manipulation of host metabolism in its benefit (Fagard et al., 2014). The relevance of N recycling, transport, and storage in *P. radiata* defence response against *F. circinatum* has been suggested by the enrichment of several lncRNAs related to allantoin metabolism (Zamora-Ballesteros et al., 2022). On the other hand, the general accumulation of amino acids in *P. radiata* and *P. pinaster* after *F. circinatum* infection suggests the ability of the pathogen to manipulate hosts’ primary metabolism to favour its nutritional conditions by increasing the availability of N sources (Amaral et al., 2019a). In accordance, an upregulation of *F. circinatum* nutrient transporter genes was associated to N uptake when infecting the susceptible *P. radiata* (Zamora-Ballesteros et al., 2021). Several studies evidence the impact of N availability on *F. circinatum* infection ability. Increased amounts of N predispose pines to *F. circinatum* infection and increase the severity of PPC symptoms (e.g., Lopez-Zamora et al., 2007; Swett et al., 2016). In nurseries, this could be explained by the stimulation of succulent shoot growth which would allow the infection of young pine seedlings without wounding (Mitchell et al., 2011). Fertilization of *P. radiata* using aerated compost tea reduced *F. circinatum* concentration likely because it is rich in N-fixing bacteria which decrease the inorganic N available restricting fungi growth and sporulation (Otero et al., 2020); while the reduction of PPC incidence in *P. taeda* seedlings grown under elevated CO_2_ has been hypothesised to be related to decreased N levels and increased C-based defence compounds (Runion et al., 2010).

### Defence response in conifers: Resin, phenolics, and PR proteins

Given that conifers are often exposed to several insects and fungal pathogens during their long lifetime, these possess a strong arsenal of defence mechanisms for protection (Franceschi et al., 2005; Kolosova and Bohlmann, 2012). These include the production of toxic secondary metabolites against the biotic agent (such as oleoresin terpenoids and phenolics), of anatomical structures to store and transport these molecules, and of PR proteins. A simple explanation of each of these mechanisms is presented in the context of *Pinus* response to *F. circinatum*.

#### Increase of resin production: Traumatic resin ducts and terpenoids

One of the first lines of defence against pests and pathogens in conifers is the production of resin (or oleoresin) in the resin ducts (Franceschi et al., 2005), representing both a mechanical and chemical defence mechanism (Kolosova and Bohlmann, 2012). Although resin ducts are constitutively distributed throughout the plant, new resin ducts (traumatic resin ducts) may be formed after pathogen attack or application of the defence elicitor MeJA to further increase conifer defence capacity (Franceschi et al., 2005; Kolosova and Bohlmann, 2012; Celedon and Bohlmann, 2019). Several studies explored the role of resin in PPC response as this is one of the most characteristic symptoms of the disease.

Although increased resin production is observed in infected trees, *F. circinatum* has shown to have the ability to grow in both constitutive and traumatic resin ducts in the susceptible *P. radiata* and use them in its favour to colonise regions of the host far from the infection site through the spread of conidiophores and conidia (Martín-Rodrigues et al., 2013). Slinski et al. (2015) reported that *F. circinatum* showed greater tolerance to *P. radiata* resin and to isolated monoterpenes than the non-pathogenic *Fusarium temperatum* Scaufl. & Munaut. Moreover, the application of MeJA as an elicitor of plant defence in *P. pinaster* led to an increase in resin duct density and thus failed to confer resistance against *F. circinatum* infection (Vivas et al., 2012), and the increased resistance of young *P. patula* seedlings to PPC using chitosan as elicitor does not seem to be associated to the synthesis of terpenoids (based on the downregulation of *DXS1*, involved in its biosynthesis) (Fitza et al., 2013). In accordance, Amaral et al. (2019b) hypothesise that the activation of the JA-dependent signalling pathway in *P. radiata* and *P. pinaster* may increase traumatic resin ducts formation and facilitate stem vertical colonization.

However, the decrease of PPC severity after wounding pre-treatment (artificial or caused by the bark-feeding PPC vector insect *Tomicus piniperda* L.) was associated to increased resin flow and/or terpene accumulation (Kim et al., 2010; Lombardero et al., 2019), and the enhanced resistance of *P. radiata* to *F. circinatum* infection after aerated compost tea fertilization was associated to the overexpression of a gene involved in terpenoids biosynthesis (*hydroxymethylglutaryl-CoA reductase 2*; Otero et al., 2020). Also, a general overexpression of genes related to terpenoids biosynthesis was observed in the resistant *P. pinea* upon *F. circinatum* infection (Zamora-Ballesteros et al., 2021). Despite this, it seems that the typical constitutive and induced resin production-associated defence responses in conifers fail to fight *F. circinatum* infection, which is able to resist terpenoids activity and take advantage of the formation of traumatic resin ducts to further colonise the plant. Moreover, the excessive production of resin during PPC development may lead to the hampering of plant water flow and result in plant mortality.

#### Phenylpropanoids pathway: The swiss knife of plant secondary metabolism

Phenolics are important constitutive and induced components of conifer defence against fungal infection (Franceschi et al., 2005), including flavonoids (such as anthocyanins), lignin, lignans, stilbenes, condensed tannins, and SA (Pascual et al., 2016). These are produced through the phenylpropanoids pathway, which is initiated by phenylalanine ammonia lyase (PAL) and represents the greatest sink of primary metabolism (Kolosova and Bohlmann, 2012; Pascual et al., 2016). ‘Non-structural’ phenolics such as flavonoids often have a direct toxic effect on pathogens (phytoalexins) and offer protection against the oxidative stress resulting from the activation of defence against pathogens through ROS signalling (Grace, 2005). The activation of this pathway and associated defences has been extensively studied in PPC.

The overexpression of genes involved in the phenylpropanoids pathway, including *pal*, was associated to the resistance of a *P. radiata* genotype (Donoso et al., 2015; Carrasco et al., 2017) and of *P. pinea* (Zamora-Ballesteros et al., 2021) to PPC; as well as to the onset of induced resistance to PPC in seedlings of *P. radiata* fertilised with aerated tea compost (Otero et al., 2020) and of *P. patula* treated with chitosan (Fitza et al., 2013). In accordance, the application of chitosan avoided the reduction of both total phenolic content and radical scavenging activity verified in *P. sylvestris* upon inoculation with *F. circinatum* (Silva-Castro et al., 2018a). In addition, early lignification and production of lignans at the infection site to avoid the spread of *F. circinatum* has been proposed to confer resistance to PPC in a *P. radiata* genotype showing increased expression of *phenylcoumaran benzylic ether reductase* (*pcber*; involved in the synthesis of secondary metabolites such as lignans) (Donoso et al., 2015) and in *P. pinea* (Zamora-Ballesteros et al., 2021). This response may protect the host against the early oxidation of phenolic compounds (e.g. lignin) by *F. circinatum* laccases (Muñoz-Adalia et al., 2016; Zamora-Ballesteros et al., 2021). An early overexpression of *pinosylvin synthase* (*pst*) was also observed in this resistant *P. radiata* genotype, which is related to the synthesis of one the most common stilbene phytoalexins in conifers (Kolosova and Bohlmann, 2012). The role of lncRNAs in the transcriptional regulation of genes involved in the phenylpropanoids pathway has been recently described in *P. radiata* after *F. circinatum* inoculation, highlighting the regulatory mechanisms behind cell wall remodelling and lignification processes upon pathogen attack (Zamora-Ballesteros et al., 2022).

Therefore, the induction of the production of several phenolics through the phenylpropanoids pathway seems a key on determining PPC resistance. Only Visser et al. (2015) reported a downregulation of PAL transcripts in *P. patula* after *F. circinatum* inoculation and Donoso et al. (2015) did not found changes in *chalcone synthase* expression (*chs*; first pathway-specific step in flavonoids biosynthesis) between a PPC resistant and susceptible *P. radiata* genotypes; but both authors hypothesise that the sampling times considered were not adequate to evaluate these responses. Although an upregulation of *pal* has been observed in needles of pine species with contrasting susceptibilities to PPC, the importance of increased phenolics contents (such as anthocyanins) to resist *F. circinatum* has been suggested both upon inoculation, at the constitutive level and after the application of phosphite as a resistance elicitor (Cerqueira et al., 2017; Amaral et al., 2019a,b; Leitão et al., 2021). Furthermore, proteomic studies hypothesised that *P. radiata* secondary metabolism may also be targeted by *F. circinatum* to negatively regulate immune response (Amaral et al., 2021b).

#### Induction of PR proteins

Antimicrobial PR proteins are only induced upon pathogen attack, being associated to HR and SAR as these offer protection from further infection at the infection site and in uninfected tissues (Kolosova and Bohlmann, 2012; Jain and Khurana, 2018). The production of PR proteins is induced by (i) molecules derived from pathogens (e.g., chitin from fungal cell wall), (ii) ROS, and (iii) by SA or JA/ET defence signalling, activating PRs involved in SAR or local acquired resistance, respectively (Ali et al., 2018; Jain and Khurana, 2018). However, the signalling behind the induction of PR gene expression is still poorly understood and it is expected not to be that straight-forward for PPC given the complexity of the phytohormone signalling proposed.

The analysis of the susceptible *P. patula* and the resistant *P. tecunumanii* (low elevation provenance) transcriptomes upon *F. circinatum* inoculation allowed to identify 801 and 646 putative PR genes, respectively (Visser et al., 2018). The lower number of transcripts in *P. patula* PR5, PR2, PR9, and PR1 orthogroups could reveal transcripts that are absent in its defence response. For example, the relative expansion of the PR9 peroxidase orthogroups in *P. tecunumanii* supports a more robust cell wall reinforcement or oxidative burst response. These enzymes are involved in the polymerization of hydroxycinnamyl alcohols—a H_2_O_2_-dependent reaction-, essential for lignin polymerization and suberin disposal (Koutaniemi et al., 2007), which can potentially enhance plant resistance against pathogens (van Loon et al., 2006). The overexpression of PR9 genes has been associated to the PPC resistant response of a *P. radiata* genotype (Donoso et al., 2015), but also to *P. elliottii* and *P. pinaster* disease development (Morse et al., 2004; Hernandez-Escribano et al., 2020). Moreover, PR9 has been suggested to be a target of *F. circinatum* effectors in *P. pinea* (Zamora-Ballesteros et al., 2021).

The upregulation of the antifungal PR1 protein transcripts has been reported in (relatively) resistant PPC hosts in association with increased expression of SA biosynthesis-related genes, which supports its involvement in SAR during *F. circinatum* infection (Carrasco et al., 2017; Visser et al., 2019; Hernandez-Escribano et al., 2020; Zamora-Ballesteros et al., 2021). Moreover, the downregulation of *F. circinatum* ergosterol biosynthesis genes could increase its susceptibility to PR1 in the resistant *P. tecunumanii* (Visser et al., 2019) as these are known to bind and sequester sterols directly inhibiting sterol-auxotrophic pathogens and sterol-prototrophic pathogens with compromised sterol biosynthesis (Gamir et al., 2017).

The upregulation of PR2 β-1,3-glucanase, PR3 chitinase, and PR5 thaumatin-like protein upon *F. circinatum* infection also showed to be related to hormone signalling and suggest an effort of the host to degrade fungal cell wall increasing its susceptibility to cell lysis and plant defence molecules (Davis et al., 2002; Morse et al., 2004; Donoso et al., 2015; Carrasco et al., 2017; Visser et al., 2019; Zamora-Ballesteros et al., 2021). PR5 was associated to PPC resistance in a *P. radiata* genotype mediated by ET (Carrasco et al., 2017) and to the relative resistance of *P. pinaster* to PPC (Amaral et al., 2019a); while PR3 upregulation occurred in susceptible and resistant hosts associated to both SA or JA/ET signalling (Davis et al., 2002; Morse et al., 2004; Carrasco et al., 2017; Visser et al., 2019; Amaral et al., 2019a), and was related to the enhanced resistance of *P. radiata* to PPC through fertilization with aerated compost tea (Otero et al., 2020).

Davis et al. (2002) performed proteomic studies regarding the role of chitinase in the *P. elliottii*-*F. circinatum* interaction finding that these were overexpressed at the mRNA level in both the susceptible and resistant genotypes but only in the symptomatic susceptible genotype at the protein level. This evidenced the importance of employing proteomic studies to further explore the role of PR proteins in hosts with varying susceptibilities to PPC upon *F. circinatum* challenge. Recent needle proteomics analysis of the PPC susceptible *P. radiata* and resistant *P. pinea* did not highlighted any key PR protein in response to *F. circinatum* infection (Amaral et al., 2021b).

## Final considerations and future perspectives

**Figure 3:**
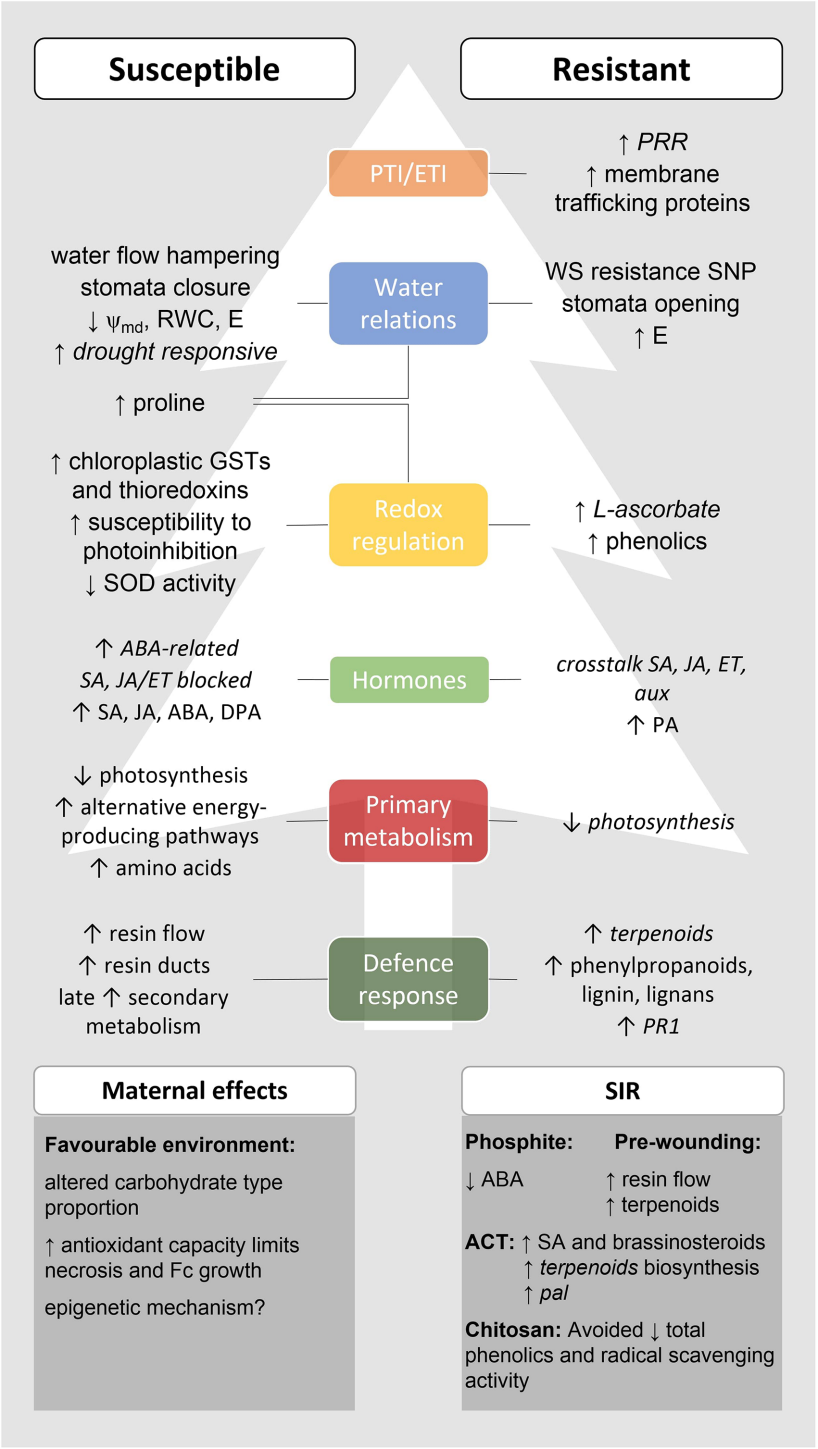
Main responses to *Fusarium circinatum* infection in susceptible and resistant hosts and effects of maternal environments and SIR in these responses. This scheme was built based on the multi-disciplinary studies focusing on pine response to PPC herein reviewed, which contributed to unveil several layers of PPC response. Italics indicate results obtained exclusively through gene expression analysis. Altogether, PPC resistant species seem to activate PTI early in the infection process and avoid *F. circinatum* effectors through membrane trafficking processes, resulting in a prompt induction of hormone signalling and defence-related responses, including the production of antioxidant compounds. Resistance to WS has been proposed as part of PPC resistance, and stomata opening and increased transpiration occurs in PPC resistant hosts upon infection although the significance of this response remains elusive. On the other hand, PTI/ETI fails in PPC susceptible pines allowing *F. circinatum* to grow inside the host and manipulate its metabolism in its benefit. The pathogen resists to the increased resin production and uses resin ducts to further colonise the host. A WS-like scenario is then established due to resin accumulation and fungal growth, leading to stomata closure (reducing water loss by transpiration), induction of drought-responsive genes, and accumulation of the osmolyte and antioxidant amino acid proline. A greater susceptibility to photoinhibition, the failure of the water–water cycle and the accumulation of chloroplastic GSTs and thioredoxins were also reported. The latter may result from fungal manipulation of host metabolism to activate alternative energy-producing pathways (e.g. respiration, aerobic fermentation, and gluconeogenesis) under photosynthesis impairment to produce amino acids as an N source. The late induction of PPC susceptible host secondary metabolism is insufficient to fight PPC. Moreover, ABA accumulation is associated to PPC susceptibility and conceivably results from fungal manipulation, which is also able to block SA and JA/ET signalling. The resistance to PPC observed in hosts originated from mother trees grown under favourable environments and after treatment with phosphite, ACT, chitosan, or pre-wounding has been attributed to changes in both primary and secondary metabolism, antioxidant capacity, and hormone signalling. ABA, abscisic acid; ACT, aerated compost tea; aux, auxins; DPA, dihydrophaseic acid; E, transpiration rate; ET, ethylene; GSTs, glutathione S-transferase; JA, jasmonic acid; PA, phaseic acid; PPC, pine pitch canker; PR, pathogenesis-related protein; PRR, pattern recognition receptors; RWC, relative water content; SA, salicylic acid; SIR, systemic induced resistance; SNP, single nucleotide polymorphism; SOD, superoxide dismutase; WS, water stress; and ψ_md_, midday water potential.

The use of a comprehensive number of physiological and Omics techniques to evaluate the response of pine species with different levels of susceptibility to PPC allowed to unravel several aspects of *Pinus-F. circinatum* interaction (see Figure 3). While susceptible hosts are generally highly responsive to pathogen infection either as a result of pathogenesis or of a late and insufficient activation of plant immune defence; resistant species seem to activate more specific mechanisms that may enable them to fight *F. circinatum*.

Besides contributing for the identification of potential pathways associated to PPC susceptibility/resistance, the studies herein reviewed pinpoint fields of research that deserve deeper investigation and may contribute for the development of innovative disease control measures. These include exploring the convergence between *F. circinatum*- and drought-induced responses (including stomata regulation), taking advantage of the advanced knowledge on tree response to water limiting conditions. Also, the importance of ABA-signalling and of several antioxidants on PPC response should be further examined based on the systemic induction of resistance through chemical priming, comprising either its direct application or the application of other compounds that influence its biosynthesis. Different application timings, methods, and concentrations should be tested on seedlings with different ages to assure broad-spectrum and long-lasting resistance against several isolates of *F. circinatum*.

The recent advent of microbiome studies in hosts with varying levels of susceptibility to PPC (Leitão et al., 2021; Romeralo et al., 2022) also represents an opportunity to identify microorganisms that impact host response with potential to be employed in biocontrol strategies, such as the development of disease suppressive soils. Other advances such as the publication of the chromosome-level assembly and methylome of the 25.4-Gb genome of *Pinus tabuliformis* (Niu et al., 2022) offer new venues for genomics studies on PPC, as well as for epigenetics which are still absent. In addition, the study of *F. circinatum* secretome and exometabolome would be key to better understand its interaction with *Pinus* species allowing to determine the secreted proteins and metabolites that favour tissue penetration and colonization in susceptible hosts. Given the complexity of the next steps proposed for studying PPC and developing effective control measures, it is required that scientists from different disciplines collaborate in order to speed up the understanding of the disease and thus contribute for improving the current forest management policies.

## Author contributions

JA and GP designed the article. JA wrote the manuscript and built the figures. All authors contributed to the article and approved the submitted version.

## Funding

This work was performed under the framework of the URGENTpine project (PTDC/AGR-FOR/2768/2014), which is supported by FEDER through COMPETE (Programa Operacional Fatores de Competitividade; POCI-01-FEDER-016785) and by national funds through the Portuguese Foundation for Science and Technology (FCT), and of the F4F—Forest For Future project (CENTRO-08-5864-FSE-000031) from Programa Operacional Regional do Centro (Centro 2020), Portugal 2020 and Fundo Social Europeu. Thanks are due to FCT/MCTES (Ministry for Science, Technology and Higher Education) for financial support to CESAM (UID/50017/2020 + UIDB/50017/2020 + LA/P/0094/2020) through national funds. FCT also supported JA (SFRH/BD/120967/2016). The Spanish Ministry of Economy and Competitiveness supported LV through the Ramón y Cajal programme (RYC-2015-17871).

## Conflict of interest

The authors declare that the research was conducted in the absence of any commercial or financial relationships that could be construed as a potential conflict of interest.

## Publisher’s note

All claims expressed in this article are solely those of the authors and do not necessarily represent those of their affiliated organizations, or those of the publisher, the editors and the reviewers. Any product that may be evaluated in this article, or claim that may be made by its manufacturer, is not guaranteed or endorsed by the publisher.
